# Multifocal Medullary and Papillary Thyroid Carcinoma Occurring as a Collision Tumor: A Case Report

**DOI:** 10.1155/crip/1510607

**Published:** 2025-09-05

**Authors:** Sayali Gadre, Raji T. Naidu, Prachi R. Gaddam, Neha Mittal, Susan Cherian

**Affiliations:** ^1^Department of Pathology, Bhabha Atomic Research Centre Hospital, Mumbai, Maharashtra, India; ^2^Department of Pathology, Tata Memorial Centre, Mumbai, Maharashtra, India

**Keywords:** collision tumor, medullary, papillary, thyroid carcinoma

## Abstract

Collision tumors are rare tumors comprising two morphologically distinct tumors within the same organ without histological admixture. Thyroid collision tumors are extremely rare. We present a case of a 64-year-old male patient with a radiologically suspicious, TI-RADS-TR4 lesion in the right lobe of the thyroid. Fine needle aspiration cytology (FNAC) from the lesion was diagnosed as medullary thyroid carcinoma. Total thyroidectomy with central and right lateral neck dissection was performed. On histopathological evaluation, a collision tumor was identified. Components of the collision tumor were multifocal medullary thyroid carcinoma (MTC) and multifocal infiltrative follicular variant of papillary thyroid carcinoma (PTC). Several hypotheses have been suggested regarding the pathogenesis of the collision tumor. Further management and prognosis of the tumor depend on the component with the higher stage and more aggressive behavior. The case report emphasizes the need for thorough sampling of uninvolved areas in the specimen for microscopic evaluation and staging of each component.

## 1. Introduction

Collision tumor is a neoplastic lesion comprised of two or more distinct cell populations that maintain distinct borders. [1] Thyroid collision tumors (TCTs) consist of two histologically and morphologically distinct tumors occurring in the thyroid with no histological admixture. These tumors are very rare and constitute lower than 1% of all thyroid tumors [2]. Approximately 70 cases of TCTs have been reported in the literature to date. The most frequent association encountered in TCTs is the synchronous presence of MTC and PTC components [3]. The PTC component of TCT, in most reported cases, is papillary microcarcinoma (m-PTC). MTC and PTC are two distinct neoplasms of the thyroid which originate from follicular and parafollicular C cells, respectively. The cells of origin, histopathological features, and prognosis of these tumors are different [4]. We present a rare case of TCT comprising of multifocal medullary thyroid carcinoma diagnosed on FNAC and multifocal infiltrative follicular variant of papillary thyroid carcinoma, which was incidentally detected after surgery.

## 2. Case Report

**Figure 1:**
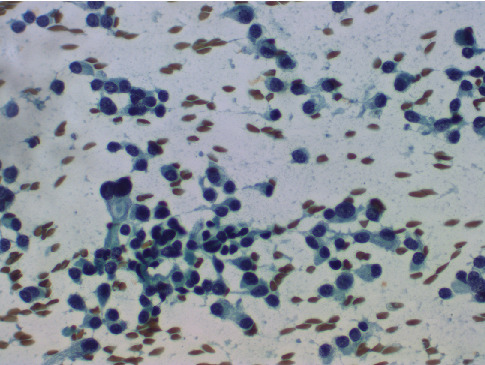
Fine needle aspiration cytology showing predominantly dissociated cells with hyperchromatic, eccentric nucleus, coarsely stippled chromatin, and moderate cytoplasm. Binucleate tumor cells are seen (Pap, ×400).

A 64-year-old male patient was detected to have a moderately suspicious (TI-RADS-TR4) lesion in the right lobe of the thyroid on ultrasonography (USG) during workup for left submandibular sialadenitis. A USG-guided FNAC was done from the same lesion and a cytological diagnosis of medullary thyroid carcinoma was given. Cytological findings revealed a dispersed population of plasmacytoid cells with moderate granular cytoplasm, eccentric hyperchromatic nuclei, and coarsely stippled chromatin (Figure 1).

**Figure 2:**
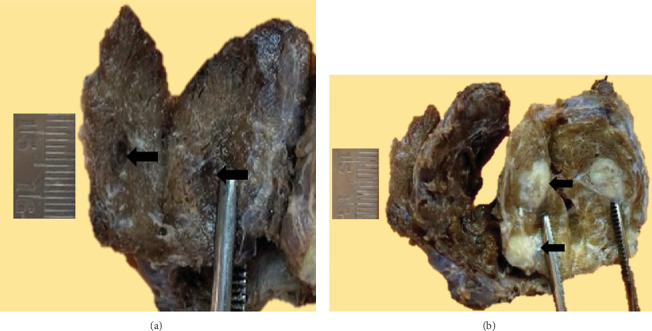
Total thyroidectomy specimen. (a) A blackish firm area (black arrow) in the right lobe of the thyroid of 0.5 × 0.5 × 0.4 cm dimension. (b) Two grey whitish firm nodules (black arrow) in the left lobe measuring 1.2 × 1 × 0.8 cm and 0.7 × 0.5 × 0.5 cm, respectively. The smaller nodule abuts the thyroid capsule.

A CT scan of the thyroid done subsequently showed an irregular, ill-defined, poorly delineated, hypoenhancing lesion in the right lobe and one hypoenhancing nodule in the left lobe of the thyroid. The serum calcitonin level performed at this time was 102 ng/L, normal range being ≤ 19 pg/mL or ≤ 19 ng/L (SI units) and ≤ 14 pg/mL or ≤ 14 ng/L (SI units). Thyroid function tests and serum parathyroid hormone level done were within normal limits. The patient underwent total thyroidectomy along with central and right lateral lymph node dissection as surgical management. On gross examination of the total thyroidectomy specimen received, a blackish firm area was seen in the right lobe measuring 0.5 cm maximum dimension (Figure 2a), and two nodules were seen in the left lobe measuring 1.2 and 0.7 cm (Figure 2b).

**Figure 3:**
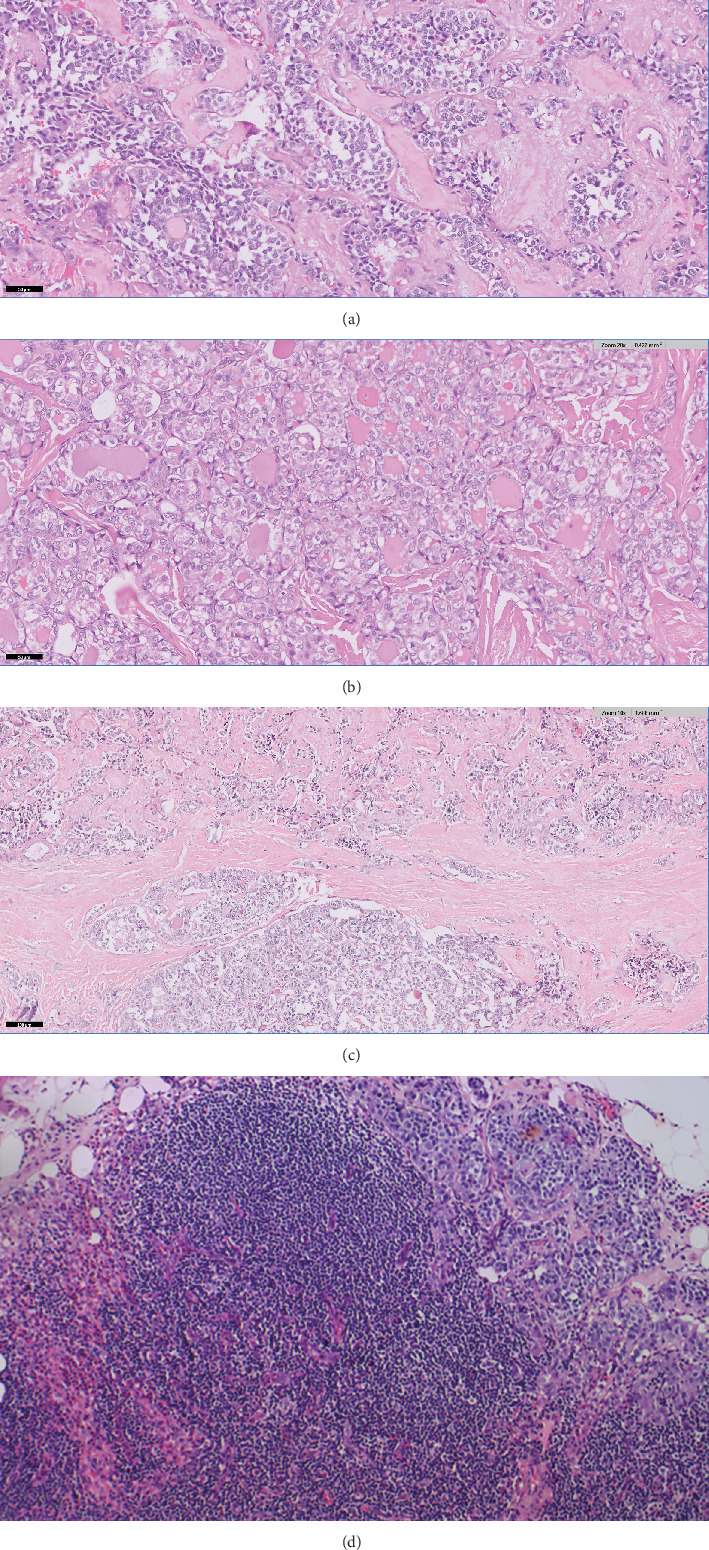
(a) Medullary thyroid carcinoma; nests and cords of tumor cells having eccentric nuclei, coarse and stippled chromatin, and moderate cytoplasm. (H and E, 200x). (b) Infiltrative follicular variant of papillary thyroid carcinoma; follicular arrangement of the tumor cells with an infiltrative pattern. Tumor cells show prominent nuclear clearing and grooves, colloid with scalloping borders (H and E, 200x). (c) Collision tumor comprising medullary thyroid carcinoma; nests and cords of tumor cells (upper half of image) and infiltrative follicular variant of papillary thyroid carcinoma (lower half of image) (H and E, 100x). (d) Metastasis of medullary thyroid carcinoma to lymph node (H and E, 200x).

**Figure 4:**
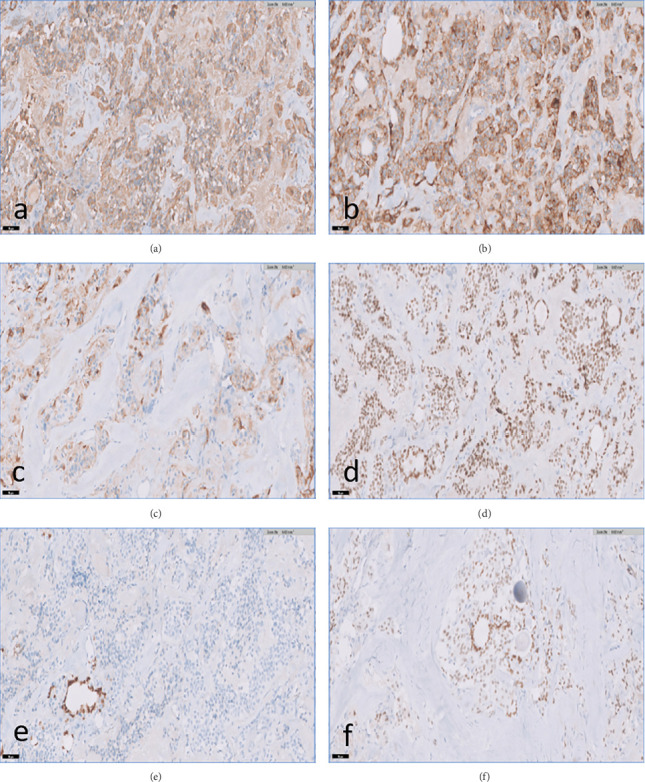
Immunohistochemistry: medullary thyroid carcinoma showing diffuse cytoplasmic positivity for (a) calcitonin, (b) chromogranin, (c) cytokeratin, and (d) nuclear positivity for TTF-1; PAX8 (with internal control) is negative in (e) tumor cells. Infiltrative follicular variant of papillary thyroid carcinoma shows nuclear positivity for (f) TTF-1.

On microscopic examination, the right lobe nodule (0.5 cm) and the left lobe nodule (1.2 cm) showed features of medullary thyroid carcinoma (Figure 3a). The left lobe nodule measuring 0.7 cm in greatest dimension showed histological features of infiltrative follicular variant of papillary thyroid carcinoma (Figure 3b). Another 0.5 cm area in the isthmus also showed features of infiltrative follicular variant of papillary thyroid carcinoma. Both the tumor components were grossly and histologically separate (Figure 3c). Microscopic extrathyroidal extension was seen in both components. There were no lymphovascular emboli or perineurial invasion. Three out of six lymph nodes from the right central compartment showed metastasis of medullary thyroid carcinoma (Figure 3d). The largest size of metastasis was 0.2 cm without extranodal extension. Right and left cervical lymph nodes received separately were free of tumor. A diagnosis of concurrent neoplasms (collision tumor) was given, comprising the following: (1) medullary thyroid carcinoma (multifocal) involving the right and left thyroid lobes with regional lymph node metastasis and (2) infiltrative follicular variant of papillary thyroid carcinoma (multifocal) involving the left thyroid lobe and isthmus. On immunohistochemistry, the MTC component was positive for calcitonin, chromogranin, TTF-1, and CK7, while thyroglobulin, TTF1, and PAX8 highlighted the infiltrative follicular variant of PTC. PAX8 was negative in the MTC component (Figure 4). The MIB1 labeling index was less than 5% in both components. On follow-up after 3 months, the patient had no complaints and the CT scan of the neck showed a clear thyroid bed and no suspicious nodes. Serum calcitonin and serum thyroglobulin levels were normal. The patient will be on three monthly follow-up to monitor for recurrence or metastases.

## 3. Discussion

Collision tumors consist of two independent neoplasms that coexist in close proximity but remain histologically separate. Mixed or composite tumors are different from collision tumors. Unlike collision tumors, composite tumors coexist intermixed within the same tumor. The source for composite tumors is the same driver mutation that induces divergent cellular differentiation [3]. To qualify for collision tumors, each tumor must be distinct and must demonstrate a definite picture of malignancy. While there is no universal classification, such tumors in an organ are often discussed based on histologic interaction, differentiation, and origin. Satter et al. proposed a classification system for dermatopathology tumors exhibiting both melanocytic and nonmelanocytic features, dividing them into four distinct categories: (1) Collision tumors consist of two independent neoplasms that coexist in close proximity but remain histologically separate; (2) combined tumors contain two intertwined malignant cell populations within a single neoplasm; (3) colonization tumors consist of a neoplasm that colonizes another tumor by growing within it, but without altering the underlying tumor's architecture (e.g., melanoma in situ permeates a basal cell carcinoma, with atypical melonocytes remaining confined to the boundaries of the carcinoma); and (4) biphenotypic tumors arise from a common precursor cell that undergoes divergent differentiation, resulting in tumor components that share overlapping immunohistochemical and molecular characteristics [5].

Collision tumors can develop in various organs, including the esophagus, stomach, lungs, uterus, kidneys, rectum, brain, and testis. Despite thyroid neoplasia being the most reported endocrine malignancy (2.1%), patients with TCTs have rarely been observed in the literature, with most TCTs consisting of papillary and medullary carcinoma [6]. In a case series of 21 cases of collision tumor, m-PTC was seen in 85.7% cases [2]. TCTs are found to be more prevalent in females at an age range of 28–84, with an increasing incidence during middle age [8]. Negura et al. has reported the mean age of presentation to be 5th to 7th decade in his case series [3].

There are many proposed hypotheses regarding the pathogenesis of collision tumor. The first hypothesis proposes that “one tumor predisposes to the other tumor”: One tumor instigates the occurrence of other tumor by alteration in the micro environment of the tumor such as blood flow, oxygen tension, and/or stem cell differentiation [7]. It is hypothesized that tumor further progresses due to presence of common carcinogenic stimulants. The second is the “divergent differentiation theory”, stating that C cells and thyroid follicles are derived from remnants of the ultimobranchial body and solid cell nests [9]. The third, known as the “stem cell theory,” suggests that collision tumors arise when stem cells have the capacity to differentiate into various cancer cell types within the same organ, or when two distinct driver mutations in shared stem cells lead to the development of two distinct tumors [2]. The last proposal is the “collision theory” suggesting that two independent tumors are located in the same lesion by simple coincidence [2]. The theory of coincidence is the most proposed theory and may justify the rare occurrence of the tumor.

Many studies in literature have reported the molecular characterization of collision tumors. Germline point mutation in RET protooncogene for MTC and point mutations of the RAS oncogene such as (N-RAS, H-RAS, and K-RAS) are reported in PTC. However, no common gene mutation has been demonstrated [10].

Imaging studies such as CT scan, MRI imaging, and PET-CT with FDG can be useful in the diagnosis of a secondary tumor. However, microcarcinoma of the PTC may not be detected by imaging in the majority of the cases; thus, thorough sampling and histopathological evaluation remain the only diagnostic tools in most cases of TCT [7]. As there is limited clinical information, the diagnosis and treatment of TCTs present a challenge to the managing physician. As reported in our case, very often, only one component of the collision tumor is recognized, and the other component is incidentally diagnosed during histopathological evaluation. Hence, during grossing and sectioning of thyroid tumors, it is important to sample the surrounding area of the known lesion to identify other unrelated lesions. Metastases to the thyroid gland are rare. The incidence is variable, up to 24% in autopsy studies, dependent on the extent of thyroid parenchymal sectioning, while clinical studies report an incidence of 2%–3% of all thyroid malignancies [7]. However, immunohistochemistry can be a useful tool to rule out infiltrating metastatic carcinoma components in a TCT. The PTC component will be positive for Thyroglobulin, TTF1, PAX8, and cytokeratin 7, whereas the MTC component will be positive for chromogranin and calcitonin.

In the case report presented, both the tumors presented as distinct nodules grossly and histologically. Although they were in close proximity microscopically, they maintained distinct boundaries with distinct immunohistochemistry findings. Hence, we reported our case as TCT with MTC and PTC components. Diagnosing TCTs is important because it directly influences the choice of treatment, for example, radioactive iodine therapy versus surgical treatment, postoperative surveillance protocols like calcitonin monitoring, and prognosis assessment. A thorough gross evaluation with extensive sampling of the specimen is recommended to avoid underreporting of cases of collision tumors in the thyroid, which will be a missed opportunity for adequate treatment and prognostication. Although the treatment of MTC and PTC collision tumors is not clearly defined, total thyroidectomy with neck dissection is the primary treatment. Follow-up with serum thyroglobulin level and calcitonin level is done. The prognosis of TCT is influenced by the component with higher stage and more aggressive behavior, which in most reported series is MTC. Sherif et al. in their case series have concluded that though collision tumors behave more aggressively than singleton tumors, metastatic and survival rates are consistent with matched singleton pathology, and no unusual difference was observed in the prognosis of patients [8]. High-risk histopathological features such as tumor focality, extrathyroid extension, lymphovascular invasion, perineural invasion, and lymph node metastasis are significant prognostically [3].

## 4. Conclusion

Collision tumors are rare tumors. Concurrent neoplasms comprising multifocal MTC and multifocal infiltrative follicular variant of PTC are extremely rare. Prognosis largely depends on the more aggressive component and high-risk histopathological features like extra thyroidal extension, lymphovascular invasion, and presence of lymph node metastases. The study emphasizes the need to extensively sample uninvolved areas and thorough microscopic evaluation for diagnosis, prognostication, and further management.

## Data Availability

All data supporting the findings of the case are contained in the manuscript.

## References

[1] Bulte C. A., Hoegler K. M., Khachemoune A. (2020). Collision Tumors: A Review of Their Types, Pathogenesis, and Diagnostic Challenges. *Dermatologic Therapy*.

[2] Thomas A., Mittal N., Rane S. U. (2021). Papillary and Medullary Thyroid Carcinomas Presenting as Collision Tumors: A Case Series of 21 Cases at a Tertiary Care Cancer Center. *Head and Neck Pathology*.

[3] Negura I., Ianole V., Danciu M. (2023). Thyroid Collision Tumors: The Presence of the Medullary Thyroid Carcinoma Component Negatively Influences the Prognosis. *Diagnostics*.

[4] Guerreiro V., Costa C., Oliveira J. (2021). Mixed Medullary-Papillary Thyroid Carcinoma With Mixed Lymph Node Metastases: A Case Report. *Clinical Case Reports*.

[5] Satter E. K., Metcalf J., Lountzis N., Elston D. M. (2009). Tumors Composed of Malignant Epithelial and Melanocytic Populations: A Case Series and Review of the Literature. *Journal of Cutaneous Pathology*.

[6] Abdullah A. M., Qaradakhy A. J., Ahmed M. M. (2022). Thyroid Collision Tumors; a Case Series With Literature Review. *Annals of Medicine and Surgery*.

[7] Bojoga A., Stănescu L., Badiu C. (2021). Collision Tumors of the Thyroid. A Special Clinical and Pathological Entity. *Archive of Clinical Cases*.

[8] James S., Sithara A., Gopinath V., Nayanar S. K. (2022). Phenotypic Appraisal of Collision Tumors of Thyroid–Initial Experience of a Rare Entity at a Cancer Centre in South India. *Asian Pacific Journal of Cancer Care*.

[9] Kim W. G., Gong G., Kim E. Y. (2010). Concurrent Occurrence of Medullary Thyroid Carcinoma and Papillary Thyroid Carcinoma in the Same Thyroid Should Be Considered as Coincidental. *Clinical Endocrinology*.

[10] Biscolla R. P., Ugolini C., Sculli M. (2004). Medullary and Papillary Tumors Are Frequently Associated in the Same Thyroid Gland Without Evidence of Reciprocal Influence in their Biologic Behavior. *Thyroid*.

